# A privacy-preserving expert system for collaborative medical diagnosis across multiple institutions using federated learning

**DOI:** 10.1038/s41598-024-73334-7

**Published:** 2024-09-27

**Authors:** S. Markkandan, N. P. G. Bhavani, Srigitha S. Nath

**Affiliations:** 1 School of Electronics Engineering (SENSE), Vellore Institute of Technology, Chennai, Tamil Nadu India; 2 Department of ECE, Saveetha School of Engineering, Saveetha Institute of Medical and Technical Sciences, Saveetha University, Chennai, 602105 India; 3 ECE, Saveetha Engineering College, Saveetha Nagar, Thandalam, Chennai, Tamil Nadu 602105 India

**Keywords:** Privacy-preserving, Federated learning, Homomorphic encryption, Medical diagnosis, Deep learning, Engineering, Mathematics and computing

## Abstract

Expert system recommendation assists the healthcare system to develop in real-time monitoring and diagnosis of patient conditions over several healthcare institutions. Privacy concerns, however, present significant problems since patient data leaks can lead to big effects including financial losses for hospitals and invasions of personal privacy for people. To address these issues, the research introduces a privacy-preserving collaborative medical diagnosis (CMD) method on a federated learning (FL). FL maintains patient privacy and data localization by spreading only model parameters, therefore enabling training models on remote datasets. The combination of Partially Homomorphic Cryptosystem (PHC) and Residual Learning based Deep Belief Network (RDBN) ensures an accurate and safe classification of patient physiological data. Experimental results show that the proposed method is successful in maintaining the diagnostic accuracy over numerous healthcare institutions and protecting privacy. The results show that the RDBN and PHC computations requires around 1000 ms and 150 ms, respectively for classification and privacy; the data transmission from the user to server and from server to user is 5 MB and 4 MB, respectively. Finally with a 30% reduction in overhead, the proposed approach offers an average increase in classification accuracy of 10% over multiple datasets.

## Introduction

The incorporation of expert recommendation systems into healthcare have revolutionized medical diagnostics by improving real-time monitoring and diagnosis of patient problems over wide healthcare institutions^1^. These expert systems increase diagnostic efficiency using accurate and rapid medical diagnosis generated from clinical datasets, where it reduces the medical expenses and provides convenient healthcare services. The implementation of such systems causes significant challenges regarding data security and privacy^2^.

Medical data is considered sensitive and hence privacy becomes crucial. Unauthorized access or data leaks of a patient having major effects including insurance company discrimination and criminal exploitation on counterfeit drugs^3^. Medical organizations are often uncertain to share their databases considering privacy concerns and the large value of their clinical data. This resistance prevents the creation of an effective expert systems requiring varied datasets for proper diagnosis. Although deep learning-based systems can incorporate significant computing costs and communication overhead, which makes it less useful for real-time applications in expert systems^4^. Maintaining computational efficiency and protecting data privacy while guaranteeing significant accuracy in diagnosis is considered challenging. The development of a privacy-preserving expert system for joint medical diagnosis among various healthcare institutions becomes a major challenge^5^.

Federated learning (FL) model is a distributed technique of machine learning where multiples devices or healthcare institutions manage the model training process. Each participant trains the model locally on its data and shares the gradients or parameters to a central server, which aggregates these updates to form a global model^6^. FL forwards the data to a central server, where raw data remains on the local devices or healthcare institutions. This lowers the likelihood of data breaches in transmission as well as in storage. Since, model settings assist to greatly reduce the risk of disclosing private information, any conceivable breach reveal the model updates without directly involving personal data. Retaining personal data on local servers and minimizing data transfer assists FL that enable companies to follow stringent data privacy policies such HIPAA in the United States and GDPR in Europe. By distributing the training process, FL makes it more difficult for attackers to compromise the entire system^7^.

Expert systems control the extremely sensitive information in healthcare including treatment plans, diagnosis results, and patient medical histories. Privacy preservation in such systems is necessary as the patients are ready to provide the medical data and use healthcare services if they trust its privacy is being maintained^8^ and this trust determines the effectiveness of medical systems. Legal and ethical obligations of healthcare experts include protection of patient privacy, hence, violating patient privacy can seriously affect the healthcare institution’s reputation and result in legal actions^9^. If the sensitive health data is leaked, it can be misused by malicious entities for financial fraud, identity theft, or blackmail. Moreover, insurance companies can discriminate the patients with pre-existing conditions based on this data^10^.

Deep learning models, especially Residual Networks and Deep Belief Networks (DBNs)^11^, are powerful tools for classification challenges in healthcare as it can detect the complex patterns in data. Therefore, these models manage the privacy concerns through the training and classification stages, where residual learning addresses vanishing gradients in deep neural networks by including shortcut connections that bypasses one or more layers. This enables the need for training the deep networks, which captures the intricate patterns in medical data. DBNs are a kind of generative models with several layers of stochastic and latent variables, which are effective for feature extraction and unsupervised learning. This structure makes it suitable for medical data with limited labeled items^12^. The residual structure facilitates the deep network training, while the DBNs captures the data distribution^13^. This expert system requires an accurate diagnosis with minimum computational and communication cost after getting trained on distributed datasets without compromising data privacy.

The main objectives of the research work involve the following: (1) To develop a novel framework that protects the patient data privacy from multiple healthcare institutions during the training and diagnosis stages. (2) To develop a secure and an efficient data sharing application across multiple healthcare institutions without revealing raw data. (3) To ensure higher classification accuracy in medical diagnoses through a modified deep learning algorithm. (4) To reduce the computational and communication overhead associated with the process of training and diagnosis. The novelty lies in the combination of FL with Partially Homomorphic Cryptosystem (PHC) based Residual Learning-based Deep Belief Network (RDBN) for a privacy-preserving collaborative medical diagnosis (CMD) system. FL develops a distributed training model on the multiple distributed datasets across different institutions, while ensuring data privacy by sharing the model parameters. PHC guarantees secured operations on the encrypted data, while maintaining the data privacy without compromising the computational efficiency. Finally, RDBN improves the diagnostic system accuracy by handling the complex medical data from various institutions.

The main contributions of the research work involve the following:

- The authors develop a novel approach that utilises adaptive FL to get trained models on distributed datasets collected across various medical institutions, and it preserves data locality and preventing raw data exposure. This addresses the privacy concerns and facilitates collaboration among multiple healthcare institutions.
- PHC ensures that the sensitive information of patient is not exposed during the process of training. PHC enables secure encrypted data computation without compromising on privacy and data leakage risk.
- The proposed RDBN improves the diagnostic accuracy based on the collected data by capturing the complex patterns in medical data, which improves the reliability of the diagnoses.
- The proposed system is designed to handle a large-scale medical data, while it ensures the scalability and real-time performance in diverse healthcare environment. The experiments are conducted on real-world datasets, such as Dermatology UCI and Early-Stage Diabetes Risk Prediction UCI, to validate the system effectiveness.

The outline of the paper is discussed below:  “Related works” section provides the related works. “Proposed method to Proposed Expert System based RDBN” sections discusses the proposed method. ““Performance evaluation” section evaluates the entire work and “Conclusion” section concludes the work.

## Related works

Recent years have seen a significant attention in the healthcare industry around expert systems while it combines the privacy preservation techniques with FL. FL offers a distributed approach to machine learning, where the models are locally trained on different data entities, while it minimizes the need for centralized data storage and mitigates the privacy risks linked with data sharing^14^. This addresses the crucial challenges like communication costs, training latency, and the vulnerability of centralized systems to single points of failure.

### Expert system using federated learning

In the client end of Health Service Provider (HSP), there is a hybrid framework aiming at achieving optimal feature selection and categorization of heart disease. Support Vector Machine (SVM) are combined in this framework with Modified Artificial Bee Colony optimisation (MABC-SVM). Training delays, communication costs, and single point of failure are solved in^15^. In privacy concerns of the HSP server, the authors used a possible fix using federated matched averaging. Using deep and federated learning, a simple sequential convolutional neural network (SSCNN) model that monitors the user data privacy and test accuracy enhancement in^16^. This work offers an architecture to effectively manage both the client and server sides using deep learning. The frontend is driven by StreamLit; the backend is built on Flower architecture. FL-based intrusion detection system (IDS) model in^17^ uses a bird swarm algorithm-based feature selection with classification (FLIDS-BSAFSC). This approach detects, identifies, and protects against intrusions using a distributed training environment. The first phase is the proposed IoT data collection in FLIDS-BSAFSC is the min-max normalisation approach. The BSA-FS technique allows to choose feature subsets and the last stage in the process of class identification is the deployment of a social group optimisation algorithm in combination with the kernel of an extreme learning machine model. Decentralised FL (DFL) in^18^ has problem-solving powers to facilitate the distributed model aggregation and the reduced reliance on a single entity. Fedstellar uses a variety of federations of real or virtualized devices to train FL models in a centralised, semi-decentralised, or decentralised fashion. Users of Fedstellar can specify a broad spectrum of possibilities as they are constructing a federation. Among the elements that might be included in this category are the type and quantity of devices used to train FL models, the network topology linking those devices, the machine learning and deep learning techniques applied, and the datasets used by every member.

### Privacy preservation based expert system

The major risk is the data breach from an unauthorised node requesting sensitive data from a cloud storage system. A privacy-preservation signaling game^19^ is used to describe the interactions occurring in IoT networks relying on edge computing. The last stage is to design a signaling Q-learning algorithm, which solves the game parameter and convergent equilibrium problems. The Improved Sensitivity Drift based k-Anonymized Data Perturbation Scheme (ISD-k-ADP) in^20^ employs a random approach to introduce a small amount of noise into the dataset, therefore simplifying the EHR data. Before transmitting this well-calibrated amount of included noise to the classification process, this method calculates the Sensitivity Drift depending on the intended level of privacy. Two Stage Bagging Pruning based Ensemble Classification (TSBP-EC), which is a part of ISD-k-ADP. TSBP-EC reduces the ensemble size using distance and accuracy-based pruning, which guarantees the ML classification. Based on blockchain technology and user privacy protections, an authentication management protocol is created in^21^. The protocol preserves identities and the parameters linked with them on a blockchain, which helps the authentication for communication healthcare organisations. To ensure the security of user login and authentication processes, the protocol combines a Chebyshev chaotic map with a three-factor authentication method. A piecewise method (PM) is created to^22^ to secure the rating values and the item sets of the users. It presents an enhanced Matrix factorization (MF) based on PM (IMFPM), which enables the global and personal information separation of item profiles. This helps us to maximise the information at our disposal. IMFPM reduces the impact of privacy noise on the degree of estimating error by a random projection technique. Further, federated self-supervised learning (FSSL)^23^ train a model collaboratively through an unlabeled data but it leads to introduction of backdoor attacks since it operates in a distributed nature. To resolve this, United Backdoor Attacks (UBA) is developed that aggregates models to significantly enhance the attack efficiency. Using trust value, federated learning is applied on virtual twins to form a Digital Twin for Mobile Networks (DTMN) that perform the model training to enhance the model reliability. This trust evaluation scheme considers direct and recommended trust, and a user behavior model considers multiple attributes of users’ to improve the privacy of the model.

**Table 1:** Summary of existing methods.

Method	Reference	Privacy preservation approach	Outcomes
Expert system using federated learning			
MABC-SVM for HSP	^15^	Decentralized aggregation	Optimal feature selection and classification of heart disease
Federated Matched Averaging for HSP	^16^	Federated learning to enhance data privacy	Detect COVID-19 from a single chest X-ray image within seconds, while ensuring data privacy
FLIDS-BSAFSC	^17^	Decentralized training to reduce privacy risks	Classify, detect, and defend against attacks in IoT datasets
Decentralized FL (DFL)	^18^	Decentralized model aggregation	Minimizes dependency on a central entity, allowing flexible training across diverse federations of devices
Privacy preservation based expert system			
Privacy-preservation signaling game	^19^	Signaling Q-learning algorithm to secure data	Achieves convergent equilibrium and practical game parameters, protecting data in edge-computing-based IoT networks
ISD-k-ADP	^20^	Sensitivity Drift-based k-Anonymized Data Perturbation Scheme	Facilitates hiding EHR data with controlled noise, enabling effective and efficient classification through Two Stage Bagging Pruning based Ensemble
Blockchain-based Authentication	^21^	Blockchain for identity storage and three-factor authentication with Chebyshev chaotic map	Ensures secure user login and authentication
Improved Matrix Factorization (IMFPM)	^22^	Piecewise Mechanism (PM) with random projection technology	Protects privacy of rating values and item sets, while reducing the influence of privacy noise on estimation error

As in Table 1, methods like MABC-SVM and FLIDS-BSAFSC improve feature selection and classification accuracy, essential for heart disease and IoT attack detection. Federated learning-based methods and blockchain protocols ensure data privacy and secure authentication. Methods like ISD-k-ADP and TSBP-EC enhance classification efficiency by minimizing ensemble size and effectively perturbing data. DFL and Fedstellar platforms support decentralized training, reducing dependency on central entities and allowing scalable model training. However, there exist several unresolved issues, where the combination of FL with scalable model architectures and advanced privacy protection mechanisms remains unaddressed. Most of the frameworks lack complete tests carried out on several datasets and in physical surroundings. Further, it should be computationally efficient to ensure safe model training in complicated data distributions.

## Proposed method

**Fig. 1:**
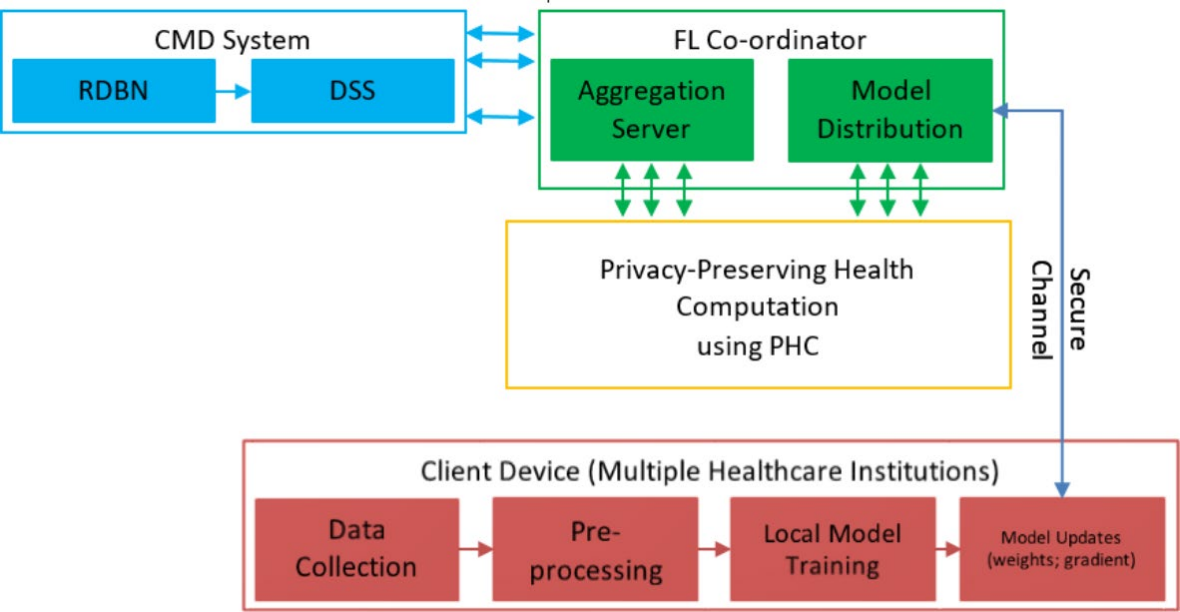
Architecture of proposed privacy preservation CMD.

The proposed privacy-preserving CMD system tackles privacy concerns and data sharing challenges among different healthcare facilities using FL. The proposed method comprises essentially in two components as in Fig. 1.

In Fig. 1, the proposed architecture for a Privacy Preservation CMD system combines several components to maintain diagnostic accuracy while preserving patient privacy across multiple healthcare institutions. At the client level, healthcare institutions handle data collection and preprocessing to clean and standardize raw medical data locally. Local models are trained on this data, incorporating privacy-preserving techniques like encryption and anonymization. Model updates, such as gradients and weights, are generated and sent to the Federated Learning Coordinator^24,25^. The aggregation server within the coordinator receives these updates, aggregates them without accessing raw data, and produces a global model update, which is then redistributed back to the client devices. To further ensure privacy, the system includes a Privacy-Preserving Health Computation using PHC module, featuring encryption and anonymization capabilities, as well as continuous monitoring of privacy metrics and differential privacy mechanisms that add noise to data or updates. At the core of the CMD system is a RDBN, a machine learning model trained using federated learning and enhanced by residual connections for improved learning. This model supports a decision support system that provides diagnostic recommendations and integrates seamlessly with local healthcare systems. Communication channels secured by robust protocols ensure the safe transmission of model updates between client devices and the aggregation server.

1. FL guarantees that raw data remains local and the model parameters are shared by distributing the learning process among different healthcare institutions, hence facilitating collaborative modeling. The research enhances the privacy and helps to lower the data leakage using this distributed technique, namely, PHC, which allows certain computations on encrypted data without decrypting it. This method ensures that sensitive information is not shared during the training procedure.
2. The proposed expert system uses RDBN algorithm to classify the patient physiological data. By maintaining privacy, the proposed DL effectively finds the complex patterns from the data, while improving diagnosis accuracy.

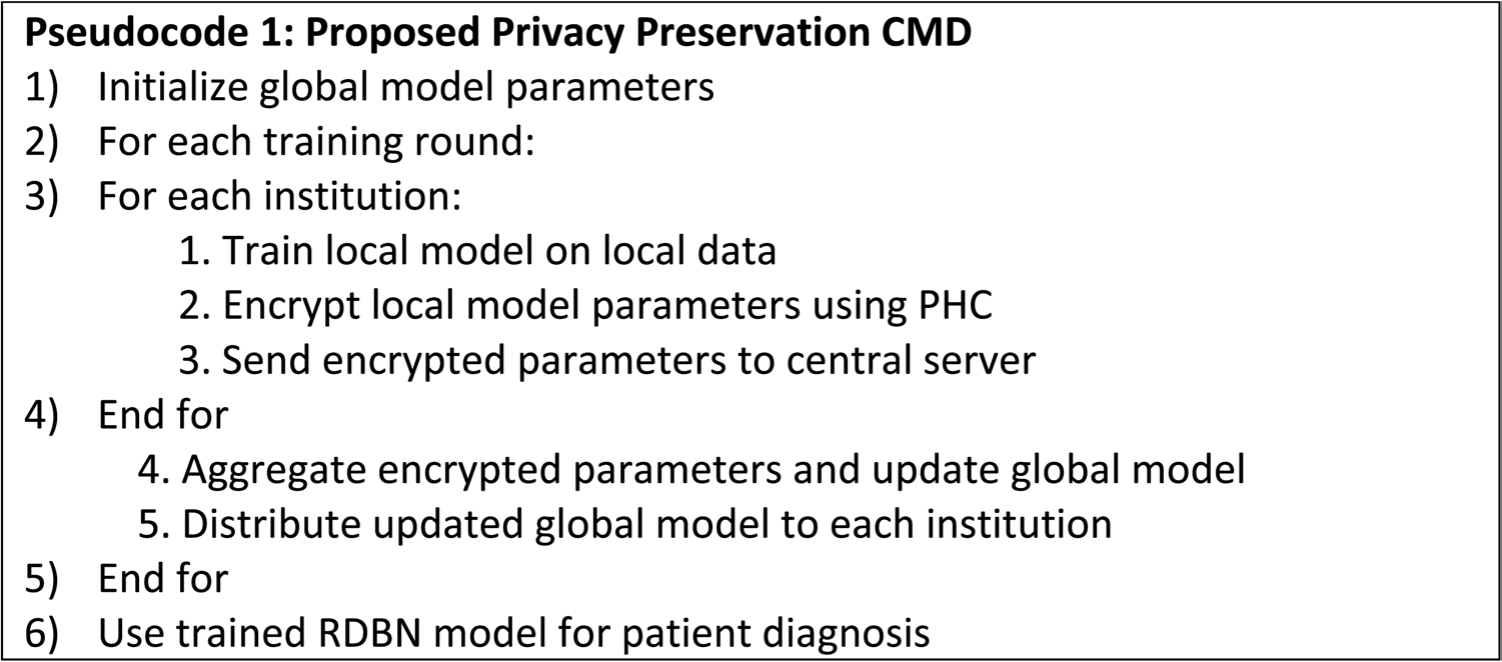


### Preliminaries

Data sharing problem among the different healthcare facilities means securely combining sensitive patient data to develop machine learning models without compromising privacy. FL addresses this by allowing healthcare institutions to cooperatively train a global model while preserving data distributed and encrypted. Decentralizing the training process assists FL to present a solution.

#### Data sharing problem-transforming data sharing into a federated learning problem

Instead of sending raw data to a central server, FL enables any institution (data owner) to train a local model on its own data. Often the model updates (gradients) are shared with the central server or coordinator, where these updates are combined to build a global model. Sensitive information thus remains local and never disseminated in its raw form, and hence it resolves the privacy issues. FL does not allow the raw data getting shared from the local institution, whereas the model updates via gradients are shared that lack critical information. By aggregating the updates of local model, the central server generates a global model using knowledge from diverse datasets without providing direct access to the data. Thus, FL reduces communication overhead unlike centralized methods since only model updates (gradients) are shared. This scalability is considered crucial for large-scale systems of healthcare involving diverse healthcare institutions.

The main challenge in sharing the healthcare data is ability to maintain the privacy of patients using its collective data for better medical insights. Each institution i.e., data owner owns the databases, but due to regulatory constraints and privacy concerns they are reluctant to share it. Conventional centralized systems face the risk of pooling the sensitive data into a location as it may get exposed. Moreover, direct data aggregation gets complicated by the privacy regulations and disparate data types. The methods for transforming the data into a federated learning problem are explained below:

Consider an institution *i*, which owns a dataset as in Eq. (1):

1 $${D_i}=\left\{ {\left( {{x_{i,j}},{y_{i,j}}} \right)} \right\}_{{j=1}}^{n}$$

where, *x*_*i, j*_ - input data (patient features) and *y*_*i, j*_ - corresponding label (medical condition).

Local healthcare institutions learn models *θ*_*i*_ on its own datasets *D*_*i*_. The research optimizes the local loss functions via Eq. (2):

2 $$\theta _{i}^{*}=\arg \mathop {\hbox{min} }\limits_{{{\theta _i}}} {L_i}\left( {{\theta _i}} \right)$$

where, ${L_i}\left( {{\theta _i}} \right)$-loss function that measures the difference between predicted and actual outcomes over *D*_*i*_.

Instead of providing raw data, healthcare institutions broadcast the model parameters *θ*_*i*_ or updates Δ*θ*_*i*_ to a central server as in Eq. (3):

3 $$\Delta {\theta _i}={\theta _i} - {\theta _{i - {\text{1}}}}.$$

These updates are aggregated at the central server to compute a global model parameter as in Eq. (4):

4 $${\theta _{new}}={\theta _{old}}+\frac{1}{N}\sum\limits_{{i=1}}^{N} {\Delta {\theta _i}}$$

where *N* - total number of healthcare institutions.

To protect privacy during parameter transmission, institutions utilize PHC encryption technique, where PHC ensures that the transmission of encrypted parameters only to the central server model changes Δ*θ*_*i*_ are finally decrypted. The approach successively executes local model updates, encrypted transmission, central aggregation, and thus updating the global model *θ* over several rounds *T*. while maintaining privacy and security, this iterative method allows the global model to learn from multiple data sources. Thus, by transforming the data sharing problem into a FL framework, these healthcare institutions can cooperate to improve the model accuracy without compromising patient privacy.

#### Preliminaries: RDBN algorithm

The RDBN is a complex neural network architecture utilizing Deep Belief Networks (DBNs) that is designed to improve the performance of classification by incorporating residual learning. This section investigates how DBNs is combed with residual learning to form a RDBN algorithm.

*Deep belief networks (DBNs)*: DBN is a type of generative model that comprises of multiple stochastic layers and latent variables. DBNs are trained layer by layer in a greedy fashion^26^. Each layer in a DBN consists of a RBM, and the network pre-trains the weights using unsupervised learning and then applies the supervised fine-tuning^27–29^. An RBM is an undirected graphical model including visible units *v* and hidden units *h*. The energy of an RBM is expressed in Eq. (5)

5 $$E\left( {v,h;\theta } \right)= - \sum\limits_{i} {{a_i}{v_i}} - \sum\limits_{j} {{b_j}{h_j}} - \sum\limits_{{i,j}} {{v_i}{W_{ij}}{h_j}}$$

where *a*_*i*_ and *b*_*j*_ - biases of visible units and hidden units, respectively, and *W*_*ij*_ - weights between them.

The objective of DBN is to minimize the energy function and hence the probability of a configuration (*v*,*h*) is given in Eq. (6):

6 $$P\left( {v,h} \right)=\frac{1}{Z}{e^{ - E\left( {v,h} \right)}}$$

where Z - partition function.

The RBM parameters are updated via contrastive divergence and after pre-training, the DBN is fine-tuned using supervised learning for weight optimization on classification. DBN stacks several RBMs such that the hidden layer of one RBM serves as a visible layer of the next. This method continues to create a deep architecture with hierarchical representations that gets captured in it. The DBN is modified under supervised learning techniques after pre-training to maximize the weights for the specific job, like classification.

*Residual learning*: Residual learning operates on the degradation problem in deep networks so that training error at higher network depth gets reduced. Instead of learning unreferenced functions, the main idea is learning the residual functions considering the layer inputs. The residual function for a layer is represented as in Eq. (7)

7 $$F\left( x \right)=\sigma \left( {Wx+b} \right)$$

The output from the residual connection is represented in Eq. (8):

8 $$y=F\left( x \right)+x$$

A residual block mitigates the vanishing gradient problem and allows the layers to learn residual functions *F*(*x*) w.r.t input x as represented in Eq. (9):

9 $$y=F\left( {x,\{ {W_i}\} } \right)+x$$

where *y* - output, *F*(*x*,{*W*_*i*_}) - residual function to be learnt, and x - residual block input.

For an input *x* passing through the residual blocks *L*, the output is represented in Eq. (10):

10 $$y=x+\sum\limits_{{l=1}}^{L} {F\left( {{x_l}} \right)}$$

where *x*_*l*_ - input to the *l*th residual block.

By stacking multiple residual blocks, the network learns effectively the deeper representations. This is useful in deep networks where direct mapping optimization is difficult.

*RDBN*: The RDBN combines the hierarchical feature learning of DBNs with the residual learning optimization. While learning deep, complex representations, the proposed hybrid approach generates a network maintaining simplicity of training and optimization.

Similar to a DBN, each layer of the RDBN is pre-trained as an RBM. Residual connections between layers are presented to facilitate the learning of residual functions and the output for a layer *l* is given in Eq. (11):

11 $${h^l}=\sigma \left( {{W^l}{h^l}^{{ - {\text{1}}}}+{b^l}} \right)+{h^l}^{{ - {\text{1}}}}$$

where σ - activation function, *W*^*l*^ and *b*^*l*^ - weights and biases of the layer, and *h*^*l*−1^ - input from the previous layer.

Each layer initiates the weights from RBMs via pre-training and hence the fine-tuning uses residual connections to learn the mapping functions. The entire network is fine-tuned using backpropagation to minimize a loss function.

#### Partially homomorphic cryptosystem (PHC)

A PHC is a type of encryption system where algebraic operations are performed on ciphertexts to generate an encrypted output, which upon decryption matches the results of operations performed on the plaintext. PHCs are useful when data privacy is of utmost importance, as in federated learning for healthcare, where sensitive patient data requires a privacy preserved training operation. Considering the addition or multiplication, PHCs are simpler and less computationally complex than a complete homomorphic design.

**Mathematical foundation**

- *Paillier cryptosystem or additive homomorphism*: A cryptosystem is additively homomorphic if there exists an operation ⊕ for any plaintexts *m*_1_ and *m*_2_ and its matching ciphertexts.

12 $$E\left( {{m_{\text{1}}}+{m_{\text{2}}}} \right)=E\left( {{m_{\text{1}}}} \right) \oplus E\left( {{m_{\text{2}}}} \right)$$

- *Multiplicative homomorphism or RSA*: A cryptosystem is multiplicatively homomorphic if there exists an operation ⊗ for any plaintexts *m*_1_​ and *m*_2_ and its matching ciphertexts *c*_1_ = *E*(*m*_1_) and *c*_2_ = *E*(*m*_2_) as in Eq. (13):

13 $$E\left( {{m_{\text{1}}}+{m_{\text{2}}}} \right)=E\left( {{m_{\text{1}}}} \right) \otimes E\left( {{m_{\text{2}}}} \right)$$

The process of PHC for maintaining the privacy of the healthcare data is expressed below:

1. *Key generation*: Each healthcare institution generates a public-private key pair (*p*_*k*_,*s*_*k*_) as in Eq. (14).

14 $$K() \to \left( {{p_k},{s_k}} \right)$$

2. *Encryption*: Each data owner encrypts the medical data using the public key as in Eq. (15).

15 $${c_i}={E_{pk}}\left( {{m_i}} \right)$$

where,

$${E_{pk}}\left( w \right)=\left( {\prod\limits_{{i=1}}^{n} {{E_{pk}}{{\left( {{w_i}} \right)}^{{x_i}}}} } \right)\bmod \,{N^2}$$

*E*_*pk*_ –Homomorphic Encryption function for Privacy, *w* - model weights, *x*_*i*_ - input data, and N - large integer, *m*_*i*_ - plaintext and *c*_*i*_ - ciphertext.

3. *Homomorphic operation*:
  *Addition*: If the PHC supports additive homomorphism, the central server can compute as in Eq. (16):
  16 $${C_{sum}}={c_{\text{1}}} \oplus {c_{\text{2}}} \oplus \ldots \oplus {c_n}={E_{pk}}\left( {{m_{\text{1}}}+{m_{\text{2}}}+ \cdots +{m_n}} \right)$$
  *Multiplication*: If the PHC supports multiplicative homomorphism, the central server can compute as in Eq. (17):
  17 $${C_{prod}}={c_{\text{1}}} \otimes {c_{\text{2}}} \otimes \ldots \otimes {c_n}={E_{pk}}\left( {{m_{\text{1}}} \times {m_{\text{2}}} \times \cdots \times {m_n}} \right)$$
4. *Decryption*: The computed result is sent back to the data owners for decryption as in Eq. (18).
  18 $${m_r}={D_{sk}}\left( {{c_r}} \right)$$

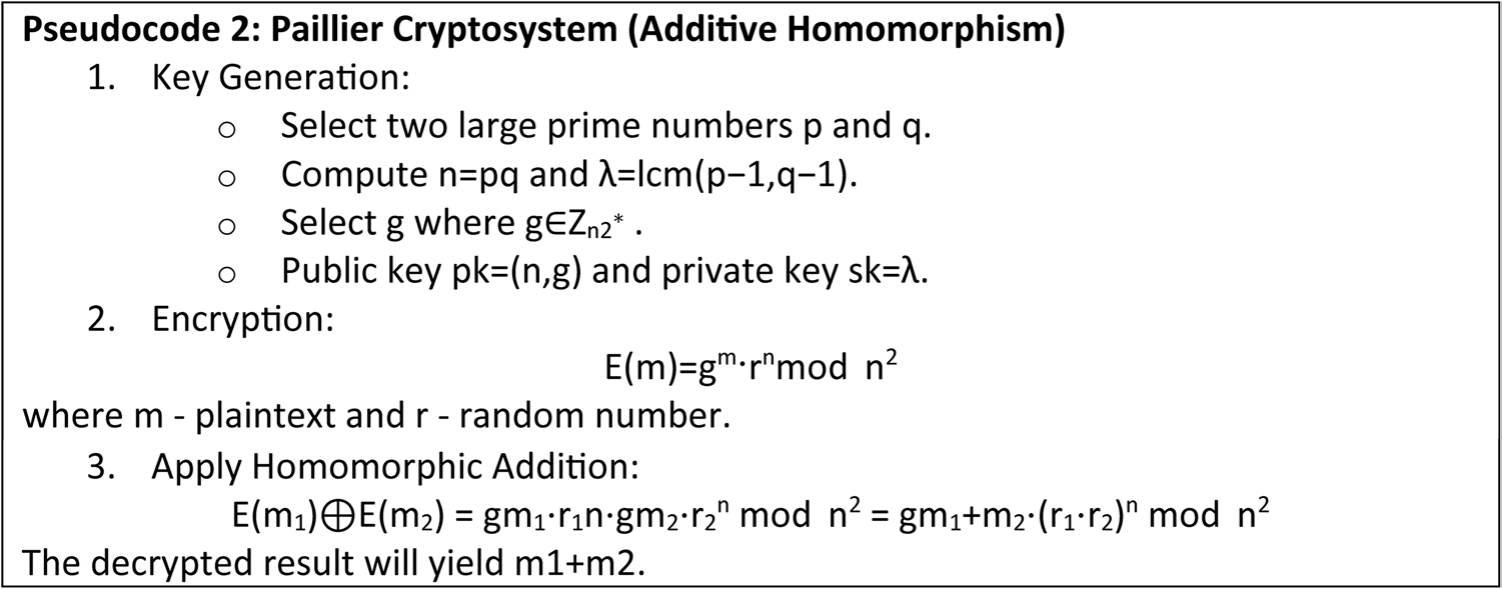


## Proposed federated learning framework

**Fig. 2:**
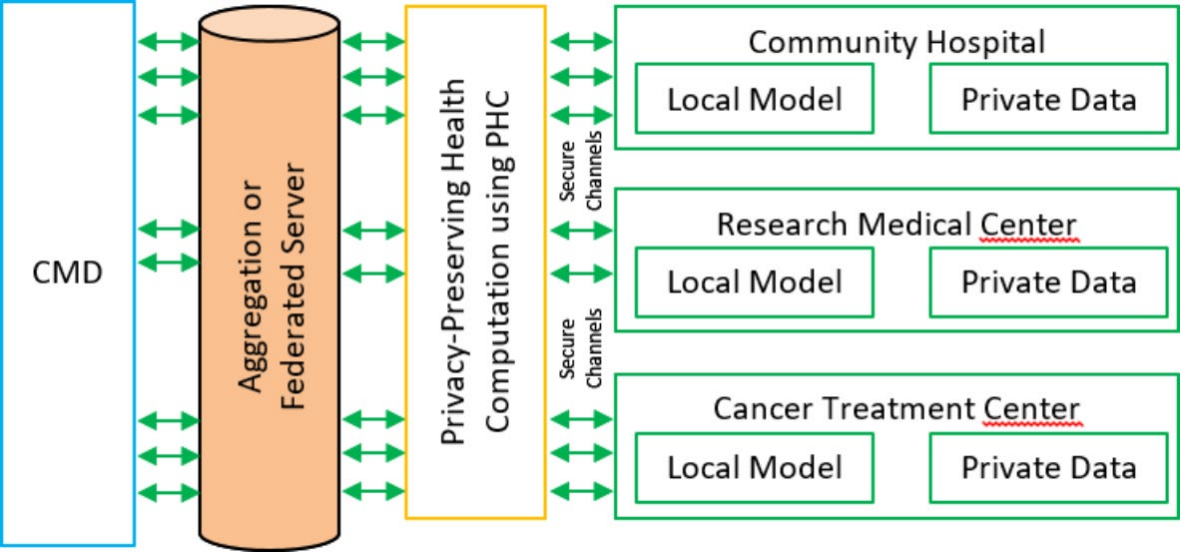
Federated learning framework.

FL, a decentralized ML approach trains the models collectively from various institutions without the need to share raw data as in Fig. 2.

The FL framework mitigates the data leakage risk significantly by offering the localized solution for each institution and it shares only the model parameters and its updates. The central server initializes the parameters *w*_0_ of global model and distributes it across entire participating institutions. Each healthcare institution *i* uses its local data *D*_*i*_​ for model training and the objective on local training for each institution minimizes the local loss of a function *L*_*i*_(*w*) as in Eq. (19).

19 $${w_i}^{{\left( {t+{\text{1}}} \right)}}={w_i}^{{(t)}} - h\nabla {L_i}\left( {{w_i}^{{(t)}}} \right)$$

where, ∇*L*_*i*_(*w*) - gradient of the local loss function w.r.t the model parameters and *η* - learning rate.

Each healthcare institution uses a PHC system to encrypt the updated model parameters *w*_*i*_^(*t*+1)^ and transfer the encrypted parameters *Enc*(*w*_*i*_^(*t*+1)^) to the central server after the process of local training with a key (*k*_*i*_) as in Eq. (20).

20 $$Enc\left( {{w_i}^{{(t+{\text{1}})}}} \right)=E\left( {{w_i}^{{(t+{\text{1}})}},{k_i}} \right)$$

The central server combines encrypted parameters from each healthcare institution to update the global model as in Eq. (21). Federated averaging is applied for the aggregation (Eq. 22) where the encrypted parameters are aggregated and decrypted to form a global model parameters.

21 $${w_i}^{{\left( {t+{\text{1}}} \right)}}=\frac{1}{N}\sum\limits_{{i=1}}^{N} {Dec\left( {Enc\left( {w_{i}^{{\left( {t+1} \right)}}} \right)} \right)}$$

where, *N* - participating institutions, and *Dec*(⋅) - decryption function.

The healthcare institutions are then provided back with the updated global model parameters *w*(*t* + 1) for the local training round and this iterative process is repeated till convergence as in Eq. (22).

22 $${w_i}^{{\left( {t+{\text{1}}} \right)}}=\left\{ {Enc\left( {w_{1}^{{\left( {t+1} \right)}}} \right)+Enc\left( {w_{2}^{{\left( {t+1} \right)}}} \right)+ \cdots +Enc\left( {w_{N}^{{\left( {t+1} \right)}}} \right)} \right\}$$

By maintaining the raw data inside the local healthcare institutions and only transmitting the encrypted model updates, FL reduces the risk of data leaking. Encryption allows the raw data to not be inferred even in case of intercepted communication.


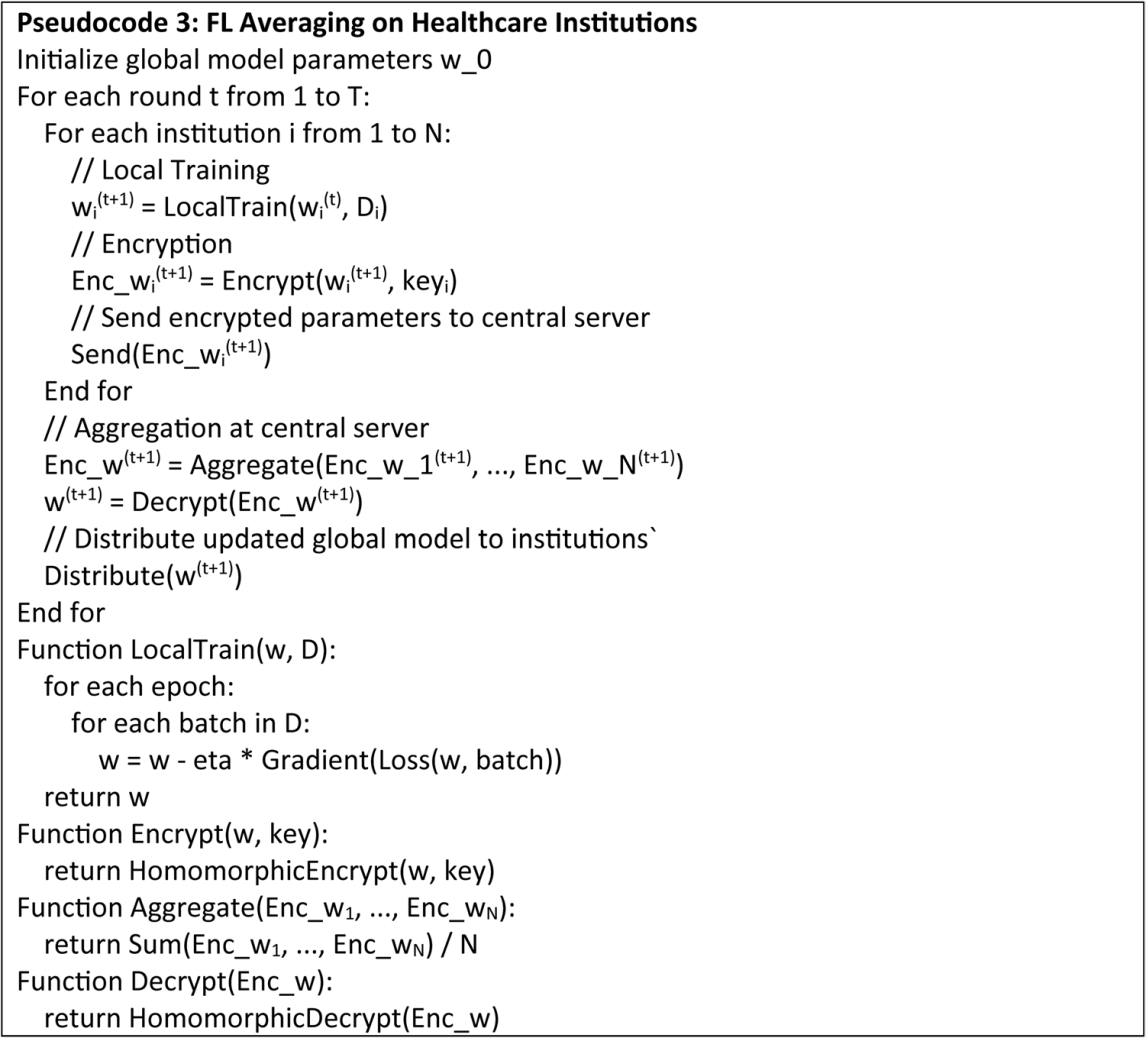


### Modeling local model parameters in federated learning

In FL, local model parameters are the trained weights and biases of ML models at each healthcare institution or a device involved in the FL process. These local parameters are crucial since they reflect the trained knowledge from any local data without directly distributing the raw data.

*Local model parameters*: Each institution (*i*) maintains a dataset as in Eq. (1). Training a ML model ${f_{{\theta _i}}}\left( x \right)$using the parameter *θ*_*i*_ will minimize a local loss of a function *L*_*i*_(*θ*_*i*_) as in Eq. (2).

*Modelling local model parameters*: Each institution starts with a ML model with a parameter *θ*_*i*_. Localizing training of the model ${f_{{\theta _i}}}\left( x \right)$on the dataset *D*_*i*_ assists to minimize *θ*_*i*_. The local loss function as in Eq. (23) quantifies the degree of data *D*_*i*_ that fits well with the model ${f_{{\theta _i}}}\left( x \right)$.

23 $${L_i}\left( {{\theta _i}} \right)=\frac{1}{{{n_i}}}\sum\limits_{{j=1}}^{{{n_i}}} {L\left( {{f_{{\theta _i}}}\left( {{x_{i,j}}} \right),{y_{i,j}}} \right)}$$

where, *L* - loss function (cross-entropy).

The parameters *θ*_*i*_ considers both the weights *W*_*i*_ and biases *b*_*i*_ of the trained model ${f_{{\theta _i}}}\left( x \right)$: *θ*_*i*_={*W*_*i*_,*b*_*i*_}, where the parameters are iteratively updated during training to minimize the losses *L*_*i*_(*θ*_*i*_).

From the datasets, consider the application of federated learning between a Clinic **B** and a Hospital **A** to develop a predictive model based on EHR. Each local institution gets trained using a neural network on its unique set of data, where Hospital **A**, trains a model ${f_{{\theta _A}}}\left( x \right)$ on its dataset *D*_*A*_​ and Clinic B, trains a model ${f_{{\theta _B}}}\left( x \right)$on its dataset *D*_*B*_. Both the models ${f_{{\theta _A}}}\left( x \right)$ and ${f_{{\theta _B}}}\left( x \right)$applies with distinct local parameters *θ*_*A*_ and *θ*_*B*_. Local model parameters *θ*_*i*_​ for two healthcare institutions shown in Table 2.

**Table 2:** Local model parameters *θ*_*i*_​ for two healthcare institutions.

Institution i	Model parameters θ_i_
Hospital **A**	*θ*_*A*_={*W*_*A*_,*b*_*A*_}
Clinic **B**	*θ*_*B*_={*W*_*B*_,*b*_*B*_}

*W*_*A*_ and *W*_*B*_ - weight matrices, and *b*_*A*_ and *b*_*B*_ - bias vectors.

## Proposed partially homomorphic cryptosystem (PHC)

**Fig. 3:**
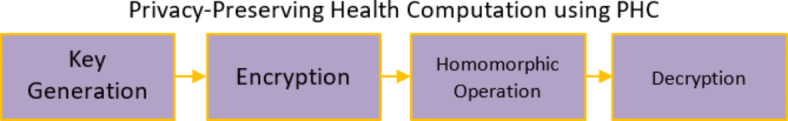
Partially homomorphic cryptosystem.

A PHC as in Fig. 3 is a cryptographic technique where specific types of computations can be performed on ciphertexts producing an encrypted output, which upon decryption, matches the result of operations executed on the plaintexts. This is useful in FL since it ensures that the sensitive data remains wholly encrypted during the training process, thereby avoiding the problem of data disclosure. Using PHC in the proposed federated learning system, each institution can encrypt its model updates prior distribution to the central server and participate entirely in local training. The server compiles these encrypted updates without decrypting and hence it preserves the confidentiality of the data.

Each institution uses a public encryption key *p*_*k*_ to encrypt its locally trained model parameters *w*_*i*_(*t* + 1) as in Eq. (24).

24 $$Enc\left( {{w_i}^{{(t+{\text{1}})}}} \right)=Enc({w_i}\left( {{{^{t}}^{+{\text{1}})}},{p_k}} \right)$$

This produces an encrypted parameters say *Enc*(*w*_*i*_^(*t*+1)^) and then it is sent to the central server. From each institution, the central server gets receives the encrypted model parameters and prior decryption, the homomorphic features of the encryption allow the server to calculate the total encrypted values as in Eq. (25).

25 $$Enc\left( {\sum\limits_{{i=1}}^{N} {w_{i}^{{\left( {t+1} \right)}}} } \right)=\sum\limits_{{i=1}}^{N} {Enc\left( {w_{i}^{{\left( {t+1} \right)}}} \right)}$$

This stage uses the additive homomorphism to allow encrypted values to be aggregated, thereby producing an encrypted output that is similar to the total number of the plaintexts. After computation of the aggregated encrypted model parameters, the server transfers the result to a trusted entity and it uses the private key *s*_*k*_ to decrypt the aggregated model parameters as in Eq. (26).

26 $$w_{{}}^{{\left( {t+1} \right)}}=Dec\left( {Enc\left( {\sum\limits_{{i=1}}^{N} {w_{i}^{{\left( {t+1} \right)}}} } \right),{s_k}} \right)$$

This decryption generates the updated global model parameters *w*(*t* + 1) without disclosing the raw data and after decryption, *w*(*t* + 1) are returned to the participating healthcare institutions for the next local training cycle. PHC guarantees the sensitive data, which is not exposed during training by the FL system. Local update encryption guarantees that no useful information about the data is deduced even in case of an adversary intercepting the communication. Further, protecting the data privacy of each healthcare institution, the homomorphic aggregation allows the central server perform necessary computations and free from plaintext access.


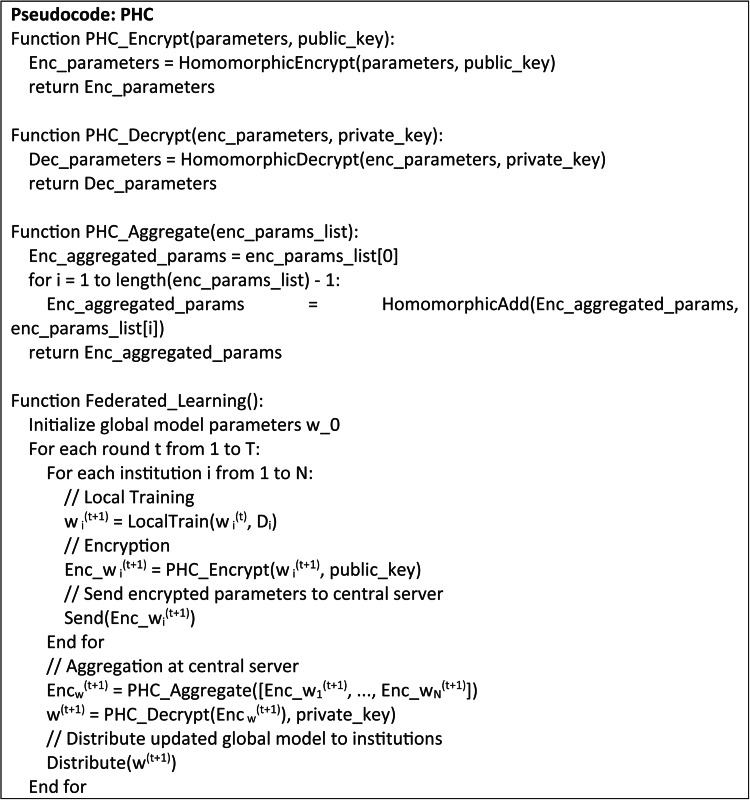


## Proposed expert system based RDBN

**Fig. 4:**
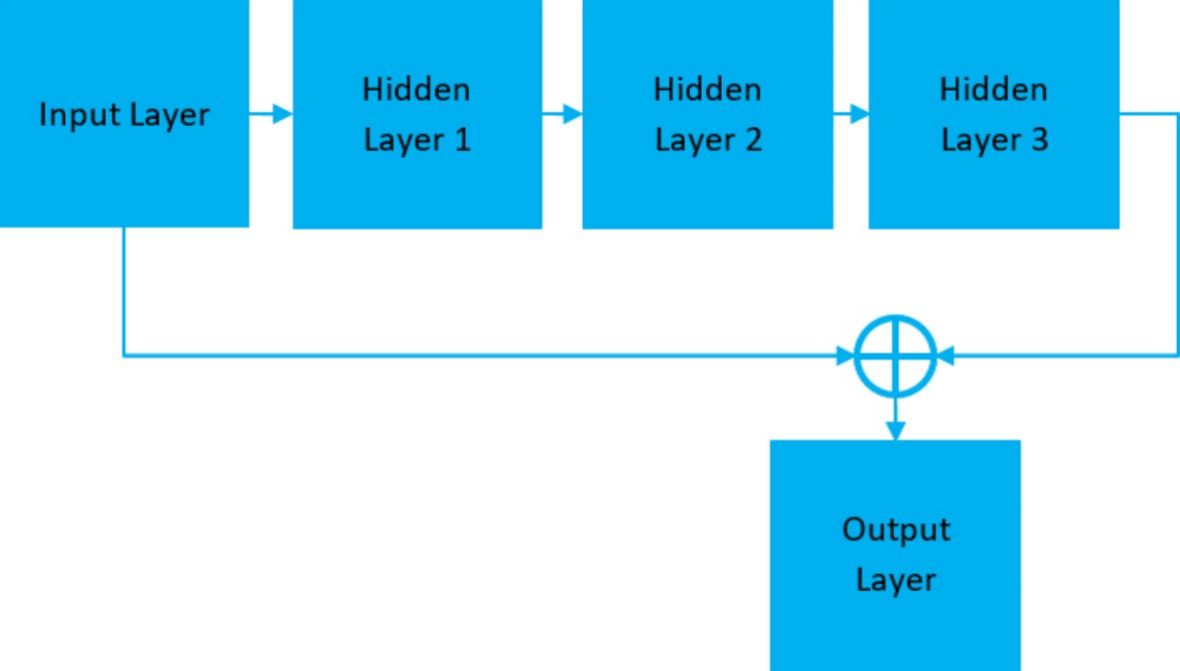
Architecture of expert system based RDBN algorithm.

The proposed solution uses the proposed RDBN in the expert system that is intended for medical diagnostics as in Fig. 4. Combining the advantages of residual learning with DBN strengths allows the system to increase the classification accuracy. The RDBN model is defined by several layers of Restricted Boltzmann Machines (RBMs) that are layered together with residual connections to support the residual learning process and prevents the vanishing gradient issue.

In the input layer, the process fed the network with patient physiological data, which is represented as a feature vector *x* as in Eq. (27).

27 $$x=\left[ {{x_{\text{1}}},{x_{\text{2}}}, \ldots ,{x_n}} \right]$$

Each RBM in the RDBN network pre-trains each layer in a greedy manner. The initial RBM learns to rebuild the input data, while the subsequent RBMs learn to train the hidden representations of the previous layer. The hidden layers are estimated as *h*^(1)^ and it is represented in Eq. (28):

28 $${h^{\left( {\text{1}} \right)}}={\text{s}}\left( {{W^{({\text{1}})}}x+{b^{({\text{1}})}}} \right)$$

where *W*^(1)^ - weight matrix of the RBM, *b*^(1)^ - bias vector of the RBM, and σ - sigmoid activation function.

The first hidden layer *h*^(1)^ is used as input for the subsequent RBM, where this process continues for all subsequent layers, and hence *h*^(*l*)^ of the *l*^th^ layer is computed in Eq. (29):

29 $${h^{\left( l \right)}}={\text{s}}\left( {{W^{(l)}}{h^{(l - {\text{1}})}}+{b^{(l)}}} \right)$$

Residual connections between the layers enable learning process and they allow the model to learn residual functions using reference to the layer inputs rather than unreferenced functions. Therefore, the residual connection between the layer *l* and (*l* + *k*) is represented in Eq. (30):

30 $${h^{\left( {l+k} \right)}}={\text{s}}\left( {{W^{(l+k)}}{h^{({\text{l}}+{\text{k}} - {\text{1}})}}+{b^{(l+k)}}+{h^{(l)}}} \right)$$

This Eq. (30) shows that the *h*^(*l*+*k*)^ incorporates the hidden representation from the layer *l* and it depends on the output of the previous layer *h*^(l+k−1)^. Following pre-training the RBMs and creating residual connections with a supervised backpropagation, which fine-tunes the whole network. The research aims to lower the classification error by varying the weights and biases across the entire network. Hence, the gradient descent helps to reduce the loss function *L*(*y*,*y*’) as in Eq. (31).

31 $${W^{\left( l \right)}}={W^{(l)}} - \eta \frac{{\partial L\left( {y,{y^\prime}} \right)}}{{\partial {W^{\left( l \right)}}}}$$

where, *y* - true label, *y*’ - predicted label, and *η* - learning rate.

For the input data, the final RDBN layer gives predicted class probabilities that directs the diagnosis decision. These probabilities are derived using a softmax activation function and it is expressed in Eq. (32):

32 $${\text{y}}^{\prime } = {\text{softmax}}\left( {W^{{(L)}} h^{{(L - {\text{1}})}} + b^{{(L)}} } \right)$$

**Algorithm: Expert System based RDBN**

1. Input the physiological data *x* into the network.
2. Pre-train RBMs:
  - Train the first RBM to reconstruct the input data.
  - Train subsequent RBM to reconstruct hidden representations of previous layer.
3. Stack multiple RBMs to form a deep belief network.
4. Add residual connections to facilitate learning.
5. Fine-tune the entire network using supervised backpropagation to minimize error.
6. Apply softmax function in final layer to obtain predicted class probabilities.
7. Determine the diagnosis.

Thus, by adding residual learning into DBN, the RDBN essentially captures the complex patterns in the physiological data and this boosts the diagnostic accuracy. It reduces the difficulties including vanishing gradients, which is suited for application in medical diagnosis.


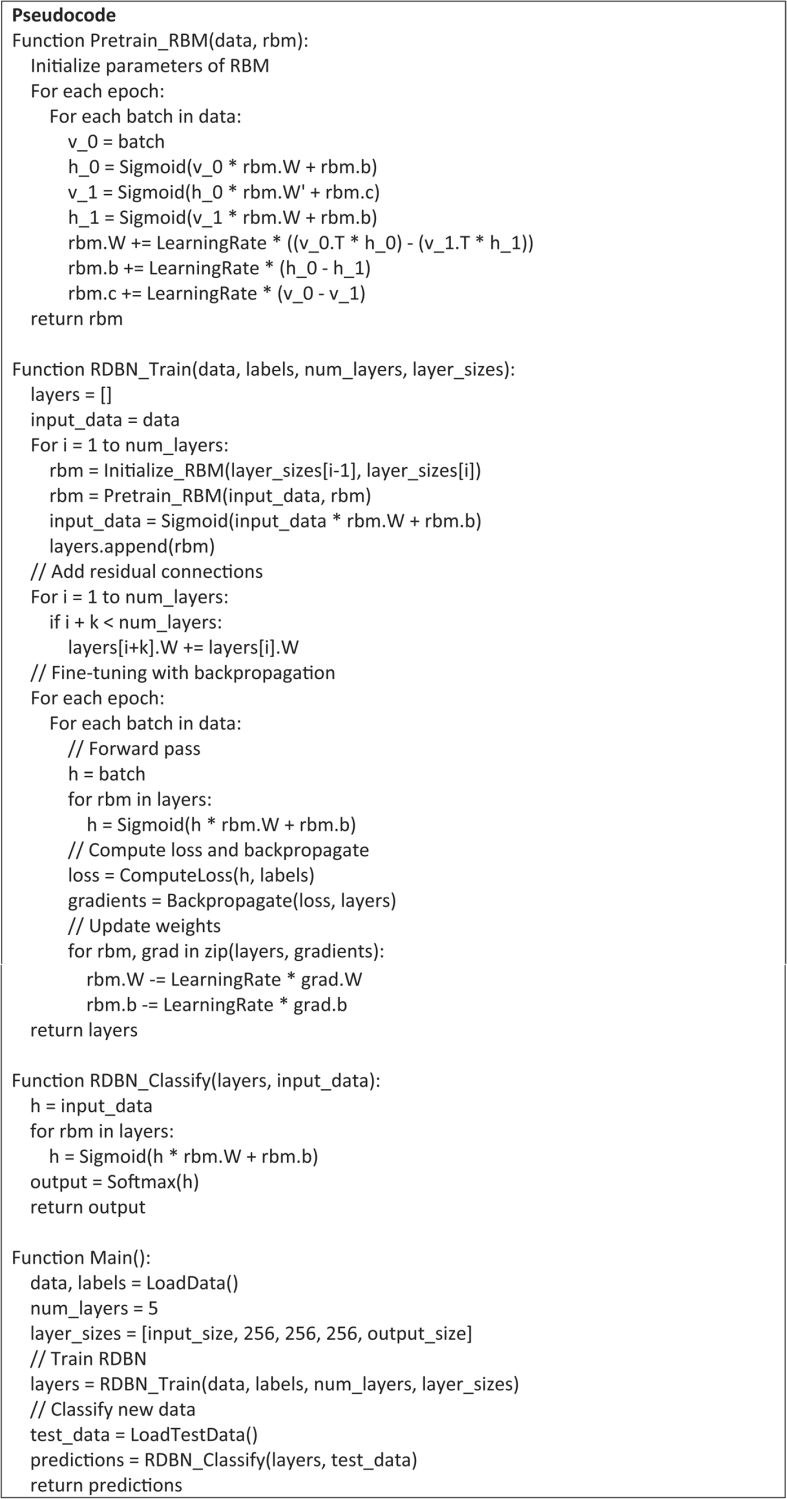


## Performance evaluation

The experimental setup utilizes a high-performance computing with a i7 processor and a 32 GB RAM to manage the computations for training the DL models. Python simulator is utilized with different libraries including TensorFlow and Github-PySyft^30^ and PyCryptodome^31^ for encryption under FL. The proposed research involves the assessment of the proposed CMD scheme against existing techniques including Improved Sensitivity Drift based k-Anonymized Data Perturbation Scheme (ISD-k-ADP), Chebyshev Chaotic Map Privacy-Preserving Authentication Management Protocol (CCM-PPAMP), and Improved Matrix Factorization based on Piecewise Mechanism (IMFPM). The experimental parameters are showed in Table 3.

**Table 3:** Experimental parameters.

Parameter	Value
Number of Hospitals	10
Number of Patients per Hospital	1000
Training Rounds	100
Batch Size	64
Learning Rate	0.001
Optimizer	Adam
Encryption Scheme	PHC
Encryption Key Size	2048 bits
Federated Learning Framework	TensorFlow
Local Epochs	5
Neural Network Architecture	Residual Learning based DBN
Number of Hidden Layers (RDBN)	5
Hidden Units per Layer (RDBN)	256
Activation Function	ReLU
Dropout Rate	0.5
Loss Function	Cross-Entropy
Noise Level for Differential Privacy	1.0
Q-Learning Learning Rate	0.1
Q-Learning Discount Factor	0.9
ISD-k-ADP Anonymity Level (k)	5
ISD-k-ADP Perturbation Level	Medium
CCM-PPAMP Chaotic Map Dimension	3
CCM-PPAMP Authentication Key Size	256 bits
IMFPM Piecewise Mechanism Segments	10
IMFPM Regularization Parameter	0.01
Communication Rounds (FL)	50
Model Aggregation Method (FL)	Federated Averaging

### Performance metrics

Each method is evaluated w.r.t its ability in preserving the data privacy, increased diagnostic accuracy, and reduced computer overhead. Performance measures used for evaluation includes accuracy, precision, recall, F1-score, and computing overhead.

1. *Accuracy*: Accuracy determines the proportion of correctly classified instances. This fundamental measure allows to determine the overall performance of the diagnostic system.
2. *Precision*: Precision among all the positive predictions indicates the proportion of true positive cases, where it defines the consistency of a positive diagnosis.
3. *Recall*: Recall shows the proportion of true positive cases among all the actual positive cases.
4. *F1-Score*: The harmonic mean of precision and recall shows a balance between the false positives and false negatives.
5. *Computational Overhead*: The computational resources including processing time and memory usage required by the system is considered critical for assessing the efficiency and feasibility of the proposed method.

Privacy and data classification analysis reveals how well the proposed system can protect data privacy under a threat scenario.

1. *False-Positive Rates*: False-positive rates monitor the incorrect identification frequency during a privacy violation. While increasing accuracy in detecting the actual violations, lowering the false-positive limits the false alarms.
2. *Reduction in Privacy Violations Over Time*: Tracking the privacy violations over time finds the impact of privacy. Effective application and improved protection of private information are revealed by a reducing trend of privacy violations over time.
3. *Full-Time Equivalent (FTE) Requirements*: Monitoring FTE requirements enables the operational cost for maintaining privacy, where reduced FTE requirements shows an efficient use of resources.
4. *Case Resolution Times*: Case resolution time is the speed with which privacy incidents are managed. Shorter resolution times increase the patient trust and helps to control any damage.

### Quantitative analysis

In this section, the proposed method is evaluated against the collected datasets from Dermatology UCI Dataset^32^ (D1), HCV Dataset^33^ (D2) and Early Stage Diabetes Risk Prediction UCI Dataset^34^ (D3).

**Table 4:** Evaluation of the proposed method against various metrics to measure it classification and privacy preserving ability.

Metric	Dermatology^32^	HCV^33^	Diabetes Risk^34^
Accuracy (%)	94.5	91.2	90.8
Precision (%)	95.0	92.0	91.0
Recall (%)	94.0	90.5	90.2
F-Measure (%)	94.5	91.2	90.6
TP	150	135	132
TN	140	130	128
FP	10	15	14
FN	15	20	22
TPR	0.91	0.87	0.86
FPR	0.07	0.10	0.09
Communication Overhead (%)	5	6	6.5
False-Positive Rates	0.07	0.10	0.09
Reduction in Privacy Violations Over Time (%)	25	30	28
FTE Requirements (hours)	10	12	12
Case Resolution Times (min)	5	7	7

Table 4 shows the results of accuracy of 94.5%, 91.2%, and 90.8% respectively for the proposed approach over Dermatology, HCV, and Diabetes Risk datasets. The results show a consistent performance in appropriately finding the cases from these datasets. Specifically for the Dermatology dataset with 95% precision and 94% recall, where the approach detects genuine positive labels while minimising false positives, and further the precision and recall are strong. F-measure balances accuracy and recall is consistently high across all datasets, where the FP and FN are low, which therefore indicating the accuracy in minimizing classification errors. The TP and TN show a strong capacity to classify the positive and negative cases. Privacy metrics show that the proposed method offers effective data handling and low error rates by maintaining low communication overhead of 5-6.5% and a low FP rate of 0.07–0.10. With a 25–30% reduction in privacy violations over time, the ability in improving privacy is viable. Case resolution times and FTE criteria shows the ability of the proposed method in offering efficient operating time and prompt issue resolution.

**Table 5:** Results of communication and computational overhead of the proposed method over different datasets.

Metric	Dermatology^32^	HCV^33^	Diabetes Risk^34^
PHC Operations (*c*_*i*_=*E*_*pk*_(*D*_*i*_) (ms)	150	200	220
FL-RDBN Computations (*C*_*i*_=*FL-RDBN*(*M*,*c*_*i*_)	1000	1100	1150
Data Transfer (User to Server) (*c*_*i*_) (MB)	5	6	6.5
Data Transfer (Server to User) (*c*_*θi*_) (MB)	4	5.5	6
FL Communication (*θ*_*g*_=⊕_*i*_*c*_*θi*_ (MB)	2.5	3	3.2

**Table 6:** Results of communication and computational overhead between proposed PHC and existing PHCs.

Metric	Dermatology^32^	HCV^33^	Diabetes Risk^34^
PHC Operations (ms)			
Proposed PHC	150	200	220
Paillier’s PHE	300	400	450
LightPHE	180	230	250
FL-RDBN Computations (ms)			
Proposed PHC	1000	1100	1150
Paillier’s PHE	1200	1300	1400
LightPHE	1050	1150	1200
Data Transfer (User to Server) (in MB)			
Proposed PHC	5	6	6.5
Paillier’s PHE	6.5	7	7.5
LightPHE	5.5	6.2	6.8
Data Transfer (Server to User) (in MB)			
Proposed PHC	4	5.5	6
Paillier’s PHE	5	6	6.8
LightPHE	4.5	5.8	6.2
FL Communication (in MB)			
Proposed PHC	2.5	3	3.2
Paillier’s PHE	3.5	4	4.5
LightPHE	2.8	3.2	3.5

The communication and computational overhead of the proposed method is evaluated across these datasets and with other existing methods including Paillier’s PHE^35^ and LightPHE^36^ in Tables 5 and 6. Operations involving encryption prior parameter sharing, accounts between 150ms and 220ms, with higher values for the Diabetes Risk dataset due to inherent complexity in PHC. FL-RDBN computations range between 1000 and 1150 ms shows a slight increase in its computational time for more complex datasets. Data transfer shows a manageable overhead of 5–6.5 MB for user to server transfer and 4–6 MB for server to user transfer. The FL communication overhead remains low, between 2.5 and 3.2 MB, which indicates efficient data parameter sharing without compromising privacy. The proposed method thus maintains a balance between communication overhead and computational efficiency, which makes it suitable for real-time applications while ensuring accurate classification and privacy preservation.

### Qualitative analysis

**Fig. 5:**
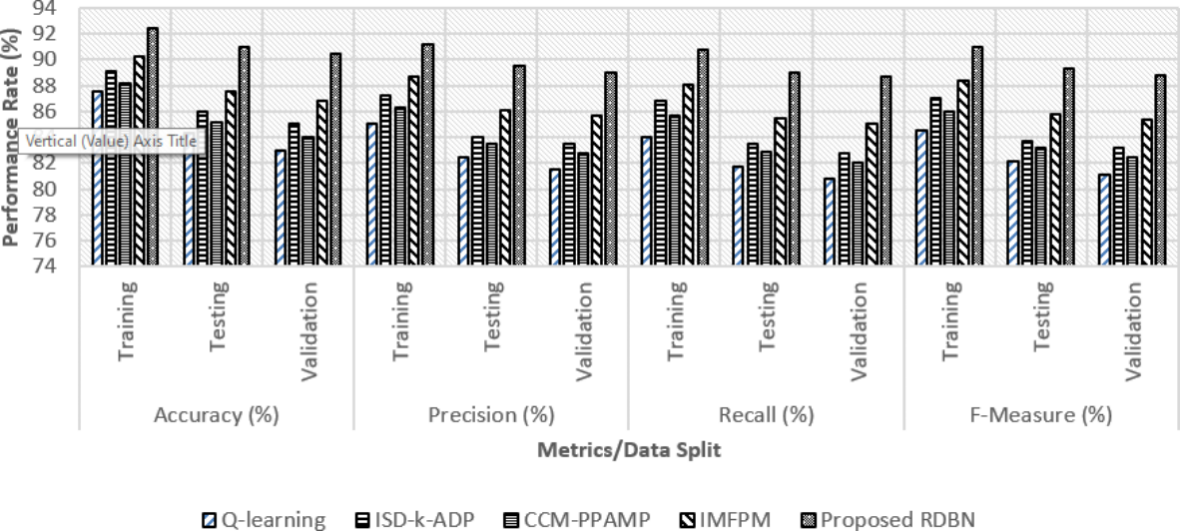
Classification performance analysis over various data split between the existing and proposed methods.

In Fig. 5, FL-PHE-RDBN achieves 92.4% in training, 91% in testing, and 90.5% in validation for accuracy, which is significantly higher than the IMFPM, CCM-PPAMP, ISD-k-ADP and Q-learning. Precision follows a similar pattern, where FL-PHE-RDBN obtains 91.2% in training, 89.5% in testing, and 89% in validation that shows its capacity to correctly identify positive cases while minimizing FPs. FL-PHE-RDBN shows its ability to detect actual positives as its scores in recall of 90.8%, 89%, and 88.7% across the training, testing and validation phases, respectively. Finally, the F-measure, which balances accuracy and recall shows that FL-PHE-RDBN achieves 91%, 89.3%, and 88.8% scores for training, testing, and validation, respectively. The proposed FL-PHE-RDBN in terms of accuracy, precision, recall, and F-measure both in training, testing, and validation phases performs better than the existing methods and this offers an accurate classification of instances with privacy constraints.

**Fig. 6:**
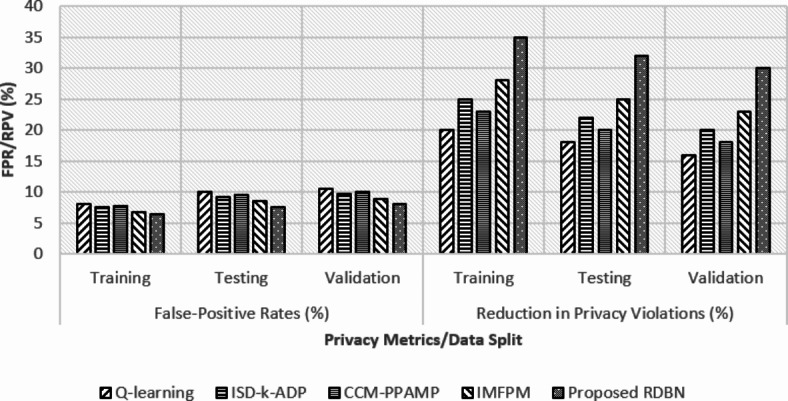
Privacy analysis using FPR and reduction in privacy violations (%) over various data split between the existing and proposed methods.

In Fig. 6, FPR and reduction in privacy violations (%) determines the privacy-preserving ability of the proposed technique. FL-PHE-RDBN shows the lowest FPR of 6.5% in training, 7.5% in testing, and 8% in validation. These rates are lesser than other methods as Q-learning shows the higher FPR at 10% in testing and 10.5% in validation phases. Maintaining system reliability depends entirely on FL-PHE-RDBN’s capacity to reduce the false alarm rates, and this reduced FPR rates of FL-PHE-RDBN shows its ability in offering better resilience. FL-PHE-RDBN outperforms w.r.t the reduction in privacy violations of 35% in training, 32% in testing, and 30% in validation than the existing methods. The consideration privacy violations reduction by FL-PHE-RDBN shows its privacy-preserving properties, hence maintaining data confidentiality and security.

**Fig. 7:**
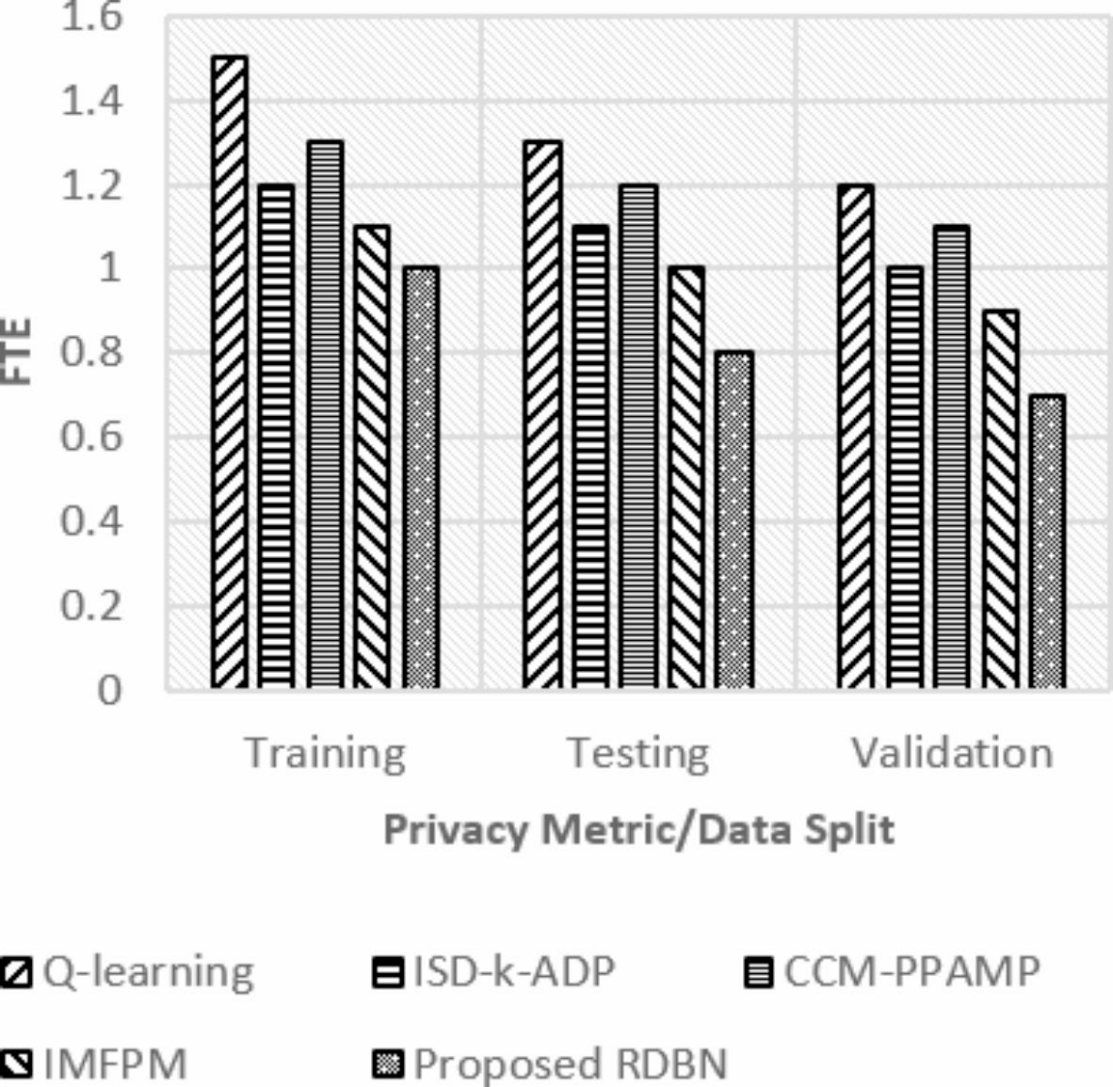
FTE requirements over different data split samples.

In Fig. 7, the FTE requirement measures labor (time)-intensiveness, where the proposed FL-PHE-RDBN shows a lower FTE with 1.0 in training, 0.8 in testing, and 0.7 in validation. This indicates that, among those involving Q-learning and other existing methods, FL-PHE-RDBN offers minimal load on the system as it needs 1.5, 1.3, and 1.2 FTEs across its respective phases. Reduced FTE criteria of FL-PHE-RDBN refer to economical and efficient deployment and maintenance.

**Fig. 8:**
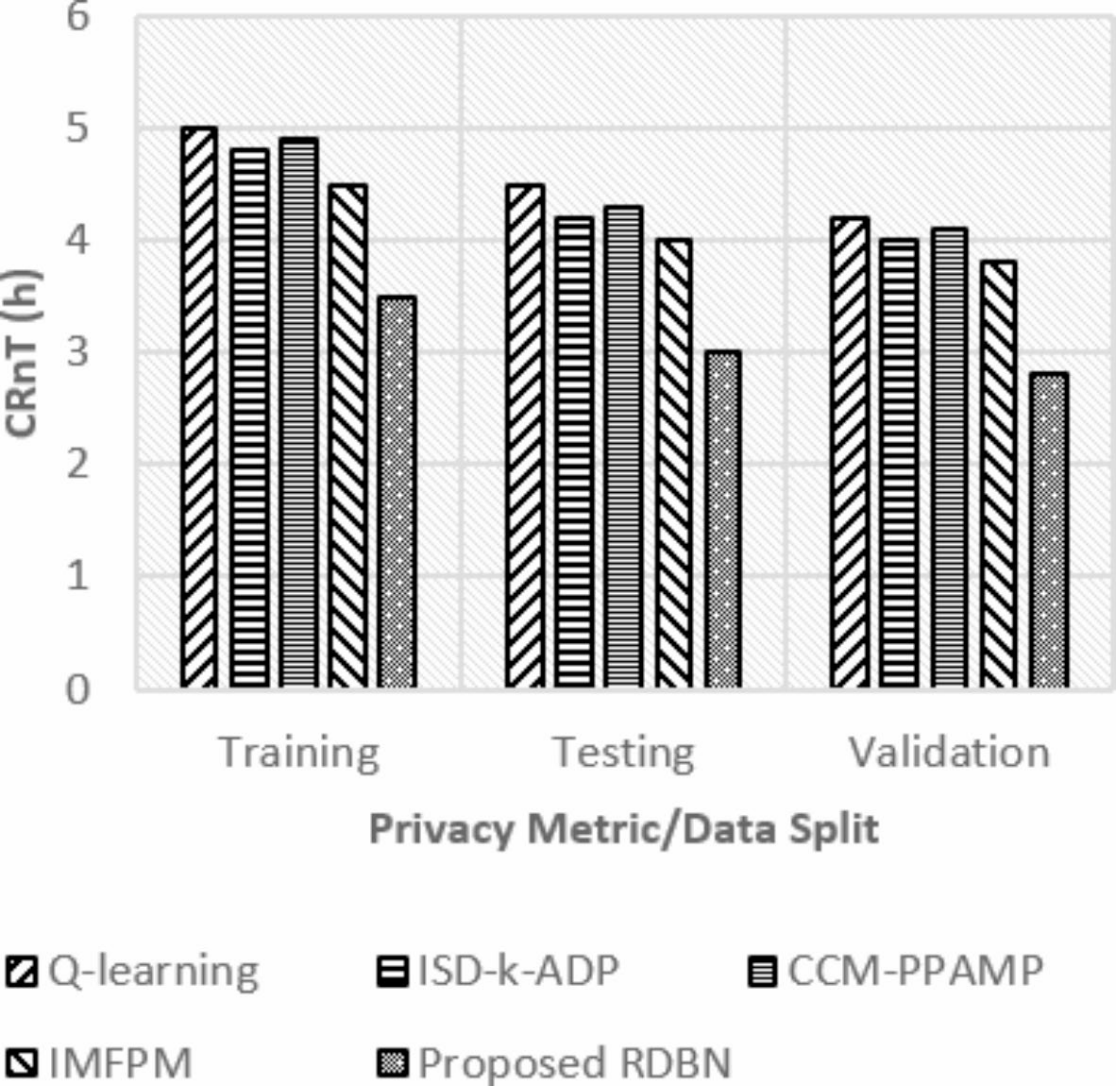
Case resolution times (hours).

Figure 8, Case Resolution Times, shows the time efficiency. The proposed FL-PHE-RDBN offers reduced resolution times with 3.5 h in training, 3 h in testing, and 2.8 h in validation. Compared with existing methods, the FL-PHE-RDBN achieves reduced resolution times, showing faster processing and decision-making ability.

**Fig. 9:**
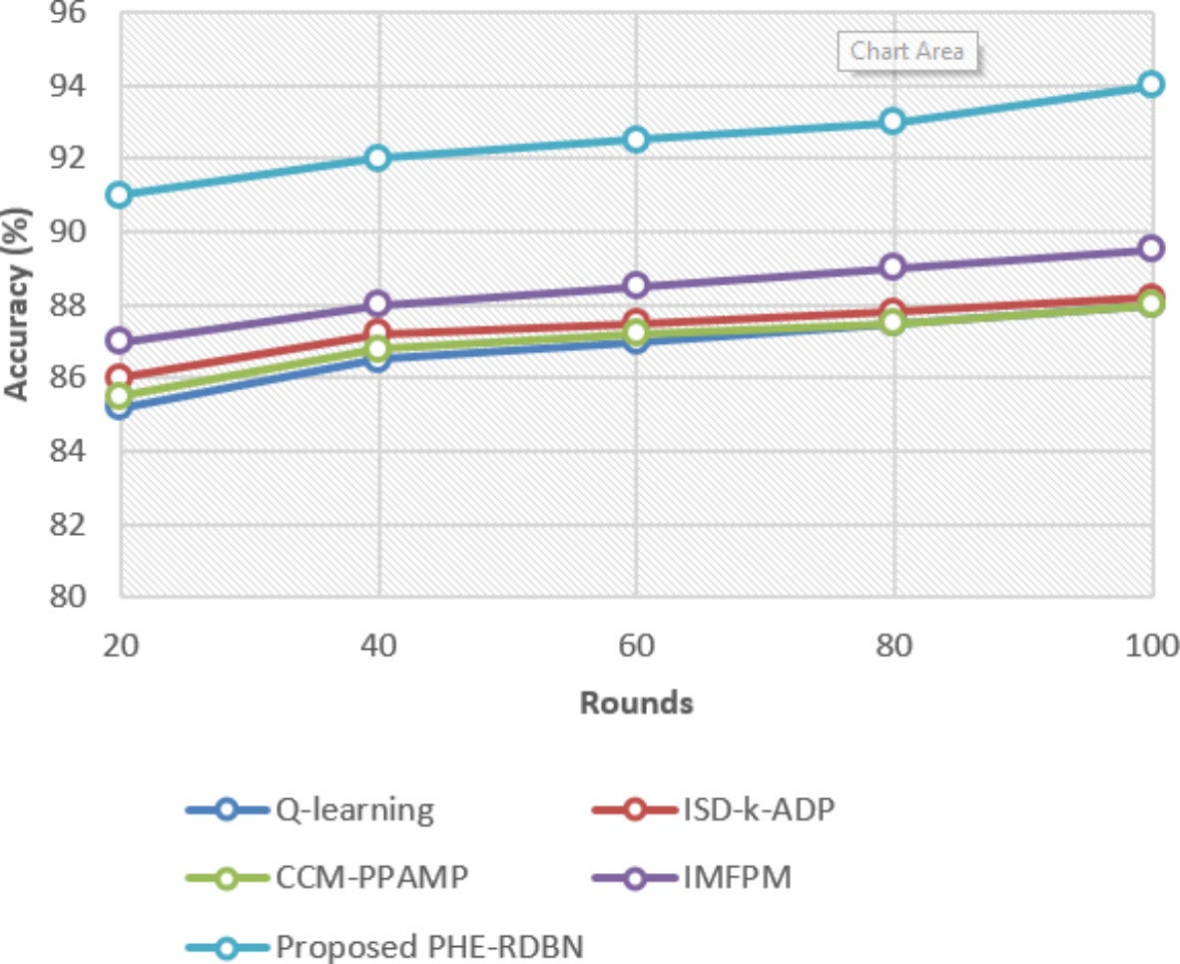
Accuracy (%) of various models over various data samples.

In Fig. 9, the proposed FL-PHE-RDBN method achieves the highest accuracy across all rounds compared to the existing methods. For instance, at initial rounds, FL-PHE-RDBN reaches 91%, surpassing existing IMFPM at 87.0% and Q-learning at 85.2%. This condition persists even at higher rounds, with FL-PHE-RDBN achieving 94% accuracy at the round final, showing its superiority.

**Fig. 10:**
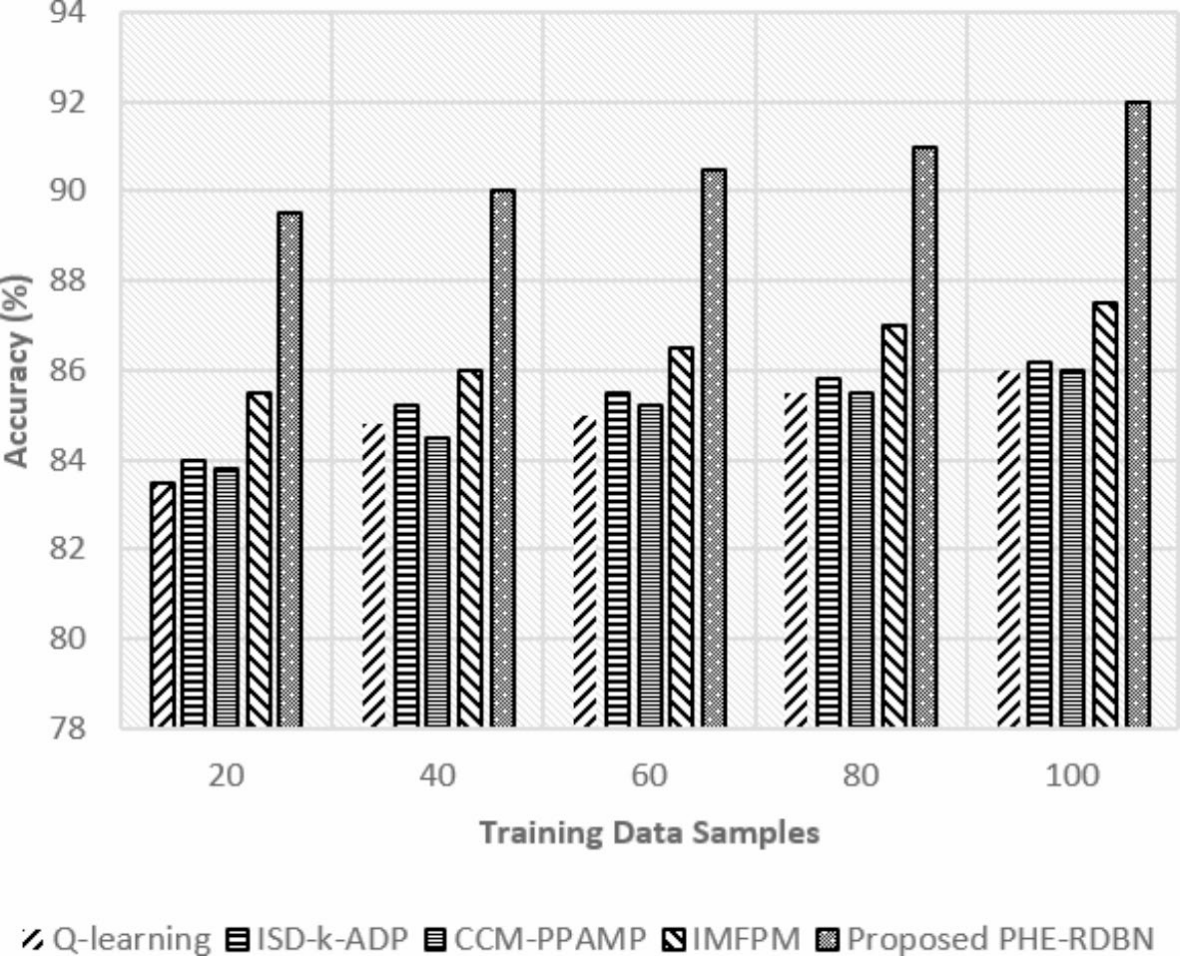
Precision (%) rate w.r.t training data samples.

In Fig. 10, at initial rounds, the precision of FL-PHE-RDBN is 89.5%, significantly higher than IMFPM (85.5%), Q-learning (83.5%), and other existing methods. While reaching the final rounds, FL-PHE-RDBN precision increases to 92%, which shows its ability to identify positive instances while minimising false positives accurately.

**Fig. 11:**
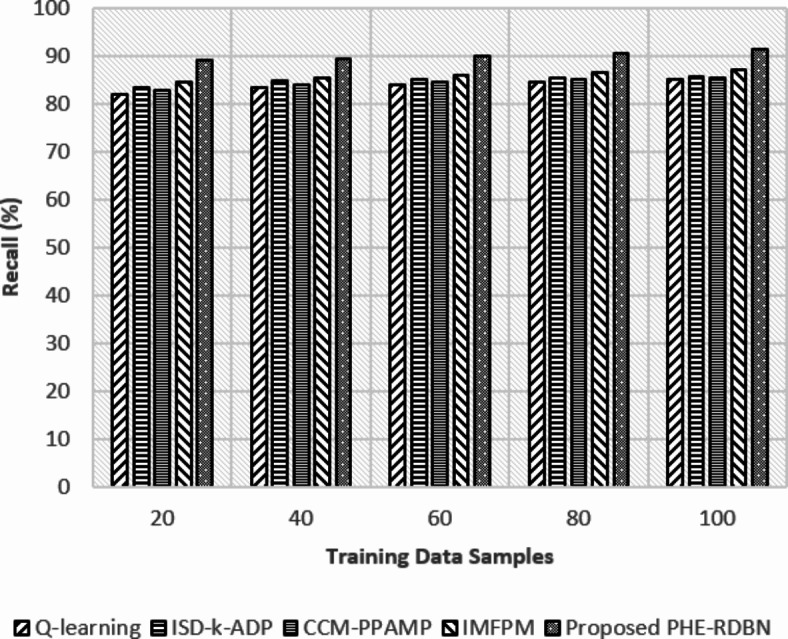
Recall (%) rate w.r.t training data samples.

In Fig. 11, considering recall, the proposed FL-PHE-RDBN achieves higher rates than other methods, where at initial rounds, its recall is 89%, compared with existing IMFPM (84.5%) and Q-learning (82) % and 91.5% at final rounds. This result shows the efficiency of FL-PHE-RDBN in finding the TP cases.

**Fig. 12:**
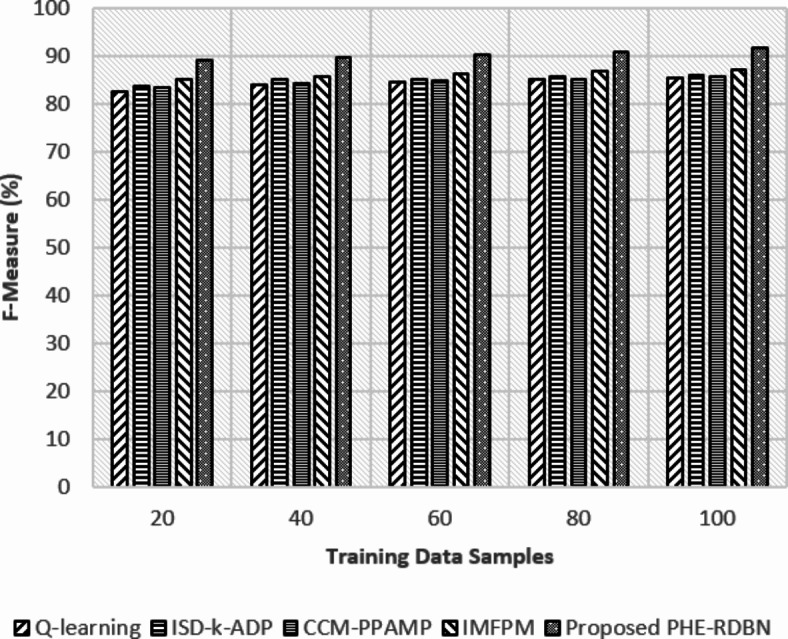
F-measure (%) rate w.r.t training data samples.

In Fig. 12, the F-measure rates balance precision and recall, where FL-PHE-RDBN at initial rounds achieves 89.2%. In contrast, the existing IMFPM and Q-learning acquire 85% and 82.7%, respectively. In the final rounds, FL-PHE-RDBN achieved a higher rate of 91.8%, which shows its robust performance.

**Fig. 13:**
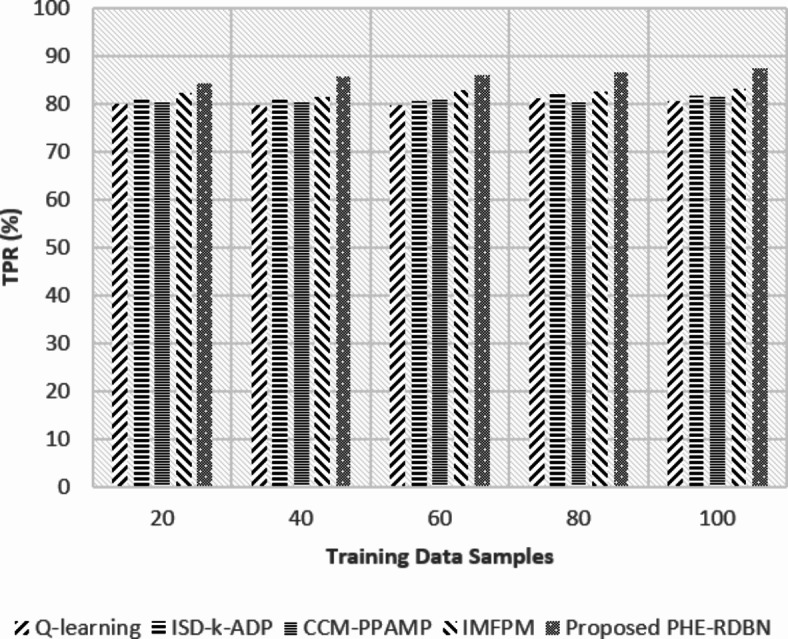
TPR (%) rate w.r.t training data samples.

**Fig. 14:**
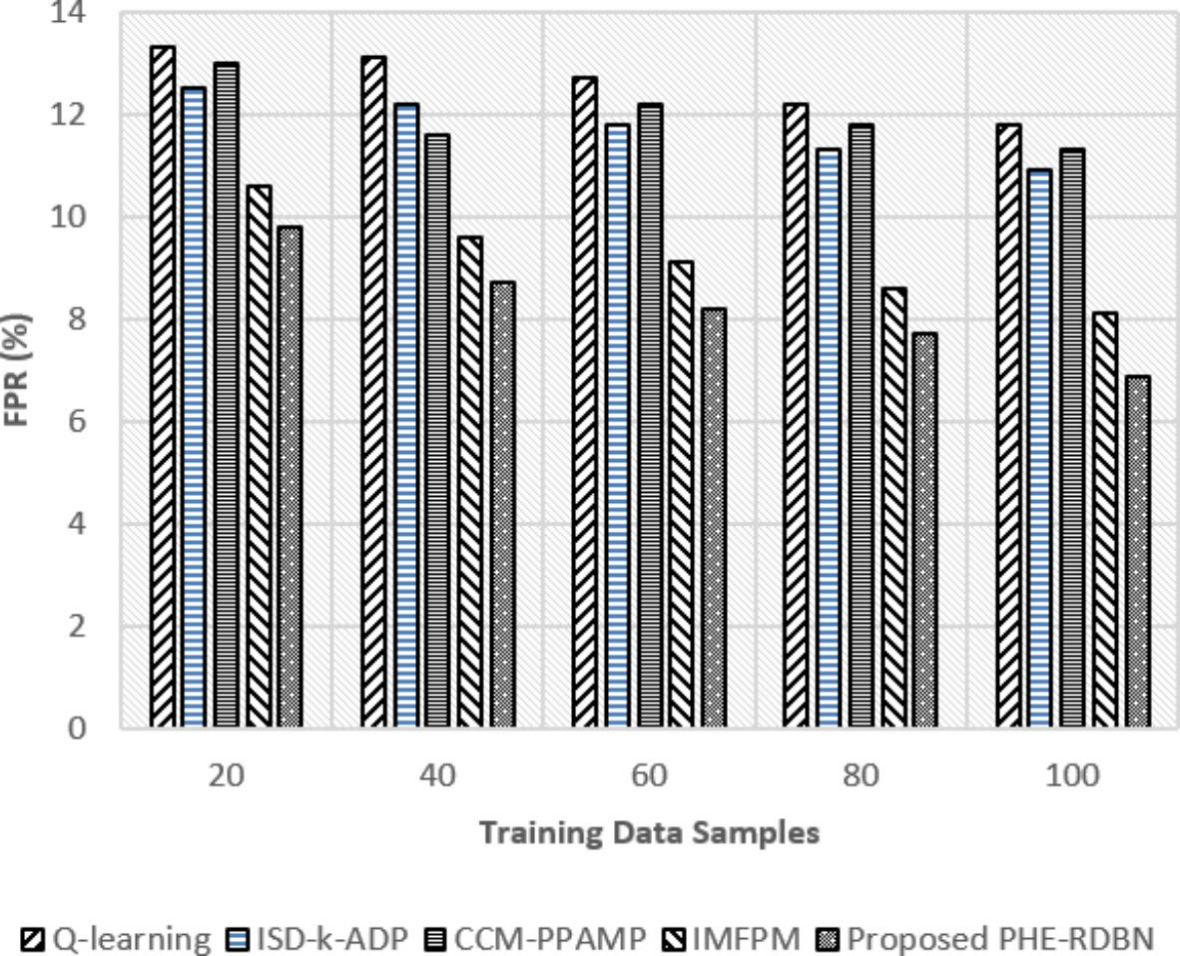
FPR (%) rate w.r.t training data samples.

FL-PHE-RDBN shows a higher TPR and a lower FPR compared to existing methods, as in Figs. 13 and 14. In the initial round, its TPR is 84.4%, and its FPR is 9.8%. In the final rounds, the TPR increases to 86.7%, and the FPR reduces to 7.7%. This shows its accuracy in TP detection and minimisation of FP.

**Table 7:** Confusion matrix.

Round	Method	TP	TN	FP	FN
20	Q-learning	700	780	120	180
	ISD-k-ADP	730	770	110	170
	CCM-PPAMP	725	775	115	165
	IMFPM	750	770	100	160
	FL-PHE-RDBN	820	740	80	140
40	Q-learning	735	745	115	185
	ISD-k-ADP	745	755	105	175
	CCM-PPAMP	740	760	100	180
	IMFPM	750	750	110	170
	FL-PHE-RDBN	850	730	70	130
60	Q-learning	725	755	110	190
	ISD-k-ADP	750	750	100	180
	CCM-PPAMP	745	755	105	175
	IMFPM	765	755	105	155
	FL-PHE-RDBN	865	725	65	125
80	Q-learning	750	750	105	175
	ISD-k-ADP	755	745	115	165
	CCM-PPAMP	750	750	100	180
	IMFPM	770	740	110	160
	FL-PHE-RDBN	800	750	110	120
100	Q-learning	755	745	100	180
	ISD-k-ADP	780	740	90	170
	CCM-PPAMP	770	740	95	175
	IMFPM	785	735	105	155
	FL-PHE-RDBN	890	730	75	85

In Table 7, FL-PHE-RDBN correctly identifies a substantial TP instance from 760 in the initial round and to 790 in the final round. FL-PHE-RDBN also maintains high TN counts, beginning at 740 in the initial round and slightly decreasing to 710 by the final round, indicating its ability to correctly identify TN instances. FL-PHE-RDBN shows the lowest FP among the methods, starting at 80 in the initial round and decreasing to 50 by the final round. FN is relatively low, with 140 in the initial round, reducing to 110 by the final round, showing its efficiency in minimizing missed positive instances. The high accuracy, precision, recall, and F-measure performance across multiple rounds, along with its superior TPR and lower FPR, shows its efficacy in expert system applications.

## Conclusion

This research developed an approach to enhance diagnostic accuracy and privacy protection in healthcare systems through FL and PHC in the CMD framework. The CMD adopts FL to enable decentralized training without raw data exchange between institutions and it mitigates privacy concerns while maintaining high diagnostic accuracy. FL ensures that each institution contributes to the learning process with their data remaining locally stored, thereby preserving patient confidentiality. PHC in CMD to enhance data security in the event of data transmission, where the data remains secure and protected from unauthorized access. In future, PHC in CMD can be improved on multiple or interdomains and the use of machine learning algorithm is suggested for privacy preservation as it speeds up the computational process.^37^

## Data Availability

The datasets generated and/or analysed during the current study are available from the corresponding author on reasonable request.
